# Antiretroviral resistance in HIV-1 patients at a tertiary medical institute in Saudi Arabia: a retrospective study and analysis

**DOI:** 10.1186/s12879-018-3339-7

**Published:** 2018-08-28

**Authors:** Maha Al-Mozaini, Tahani Alrahbeni, Reem Al-Mograbi, Abdulrahman Alrajhi

**Affiliations:** 1 Immunocompromised Host Research, Department of Infection and Immunity, The Research Centre, King Faisal Specialist Hospital and Research Centre, Riyadh, Saudi Arabia; 2 Riyadh Colleges of Dentistry and Pharmacy, Riyadh, Saudi Arabia; 3 Department of Medicine, King Faisal Specialist Hospital and Research Centre, Riyadh, Saudi Arabia

**Keywords:** HIV resistance, Antiretroviral therapy, Saudi Arabia

## Abstract

**Background:**

Since the early 90’s antiretroviral drugs have been available at King Faisal Specialist Hospital and Research Centre (KFSH&RC), a referral hospital in Riyadh, Saudi Arabia, for the treatment of both adults and children infected with HIV-1. However, up to date, there are no genetic profiling data for the resistance-causing mutations in HIV-1 virus in patients on antiretroviral drugs therapy. This paper presents an initial report and a profiling survey of drug resistance-associated mutations of **103** HIV-1 patients seen at KFSH&RC.

**Methods:**

This is a retrospective study on Patients treated at KFSH&RC since 2003 up to 2016. The analysis was done on the drug resistance mutations profiles of 103 patients who were undergoing highly active antiretroviral therapy protocols.

**Results:**

Our analysis shows that the drug resistance mutations reported in our treatment cohort of HIV-infected adults patients is similar what is internationally reported to some extent. Additionally, we have identified novel drug resistance causing mutations. Furthermore, different profile of drug resistance causing mutations was also observed.

**Conclusion:**

Patients showed both similar and new drug resistant causing mutations, early identification of these mutations is crucial to guide and avoid failure future therapy.

## Background

Antiretroviral drugs (ARV) are the group of drugs used for patients diagnosed with HIV infection. These antiretroviral are usually administered in combinations to reduce the emergence of HIV-1 drug resistance. Nonetheless, the acquisition and evolution of secondary resistance, from non-compliance is one of the major obstacles to a successful ARV therapy. Furthermore, Patients who acquire HIV-1 drug resistant viruses have fewer treatment options and are at a higher risk of morbidity [1].

The US Food and drug administration since 2012 approved 28 ARV drugs [2, 3]. These ARV drugs fall into five classes; nucleoside RT inhibitors (NRTIs), non-nucleoside RT inhibitors (NNRTIs), protease inhibitors (PIs), integrase inhibitors (II), and fusion inhibitors (FI) [4].

In general, the World Health Organization (WHO) recommended a protocol for the initial treatment of HIV-1 infection that generally includes the combined use of three antiretroviral agents. In most cases, the application of two NRTI together with a NNRTI, a PI or an II is recommended [2]. To insure a better control and outcome, a number of factors must be taken into consideration and monitored frequently such as; genotyping testing before and during ARV treatment, ARV drug resistance, individual tolerance profiles and the needs of the individual patient, as well as several interactions with other drugs. King Faisal Specialist Hospital and Research Centre, have their own protocol, which is a combination of both the WHO [5] and the international guidelines [6]. The combinations used are also based on the availability of these drugs at KFSH&RC.

The development of resistance to ARV therapy remains a major cause of treatment failure among patients living with HIV-1. Additionally, it’s also as important to measure the rates of these mutations in our populations. This will improve our understanding of resistance and lead to more refined treatment strategies and, in some cases, improves the outcome [7–9].

Antiretroviral drugs have been available at KFSH&RC, a referral hospital, in Riyadh, the capital of Saudi Arabia, for the treatment of adults and children infected with HIV-1 since the early 90’s. However, no genetic profiling of the resistance-causing mutations in the HIV-1 virus obtained from patients on ARV therapy protocols have been done nor analyzed. This paper presents an initial report and a profiling survey of drug resistance-associated mutations (DRMs) of **103** HIV-1 patients seen at KFSH&RC. Identifying these resistance causing mutations will guide us to establish an in house diagnostic assay for screening of these possible DRM as a diagnostic tool for the HIV clinic for better patient care and treatment.

## Methods

A retrospective analysis was done on the drug resistance mutations profiles of 103 patients at KFSH&RC, who were undergoing highly active antiretroviral therapy (HAART) protocols. Our selection criteria were all HIV-infected adults on HAART regimens and are experiencing virological failure (> 500 copies/mL), with resistance mutations detected by consensus sequencing. Up to date there are no commercially available genotyping test kits to monitor such mutation. Since 2003, these tests were done at our reference laboratory in Mayo clinic. Testing was performed by RT-PCR and DNA sequencing method (Trugene HIV-1 Genotyping Kit; Siemens Healthcare Diagnostics, Inc.). Results were based on manufacturer’s most recent FDA-approved interpretive guidelines. HIV-1 genotype was analyzed by TRUGENE HIV-1 Genotyping Kit (DNA sequencing assay; Bayer HealthCare LLC), and interpretive results were based on manufacturer’s Guidelines v8.0. The evaluation for DRM was at the earliest available testing done and diagnosed within 1 year of their estimated date of infection at KFSH&RC. The mutations reported and their profile for each ARV group of drugs was analyzed. The comparison was done for single drugs in addition to known combination therapy. Additionally, the percentage of primary drug resistance causing mutations reported annually was also analyzed.

## Results

This study includes 103 HIV-1-infected Saudi patients enrolled between the years 1988–2016**.** Their age ranged from 9 to 78 with a mean of 45 years. The number of males was 70 (68%) and females 33 (32%), with a ratio of male to female 2:1.

The median of CD4 count at diagnosis was 16.8 cells/μl, with an average viral load of 5.3 log10/ml. The average time (years) since diagnosis of these patients to initiation of HAART protocol 1.78 years, with an average therapy time of 14 .1 years.

All these patients had drug resistance analysis done on their viral samples, with an average of 5.9 years after the initiation of ART. The drug resistance testing for these patients was performed more than once, ranging from 1 to 12 times with a mean of 3 times in all their therapy years. Table 1 lists all the base line characteristics of the subjects.

**Table 1:** Base line characteristics of the patients in the study

Characteristic	
Age (year)	
Mean	45
Range	9–73
Gender - no. (%)	
Male	70 (68)
Female	33 (32)
Elapsed Time (year)	
Since diagnosis (mean)	14.1
Since diagnosis to initiation of HAART	
Mean	1.78
Median	0.5
Range	0–10
Since diagnosis to drug resistance testing	
Mean	5.9
Median	6
Range	0–19
No. of drug resistance testing done /patient	
Mean	3
Median	3
Range	1–12
Laboratory variables	
CD_4_ –Percentile (%)	
Mean	17.8
Median	16.8
Range	1–60
HIVQ –Log10	
Mean	5.3
Median	4.5
Range	1.7–6.4

Mutations were analyzed in relation to demographic, clinical, and laboratory variables at time of genotyping. The drug resistance is evaluated through genotyping tests. One hundred and three subjects were analyzed for mutation codon analysis.

**Fig. 1:**
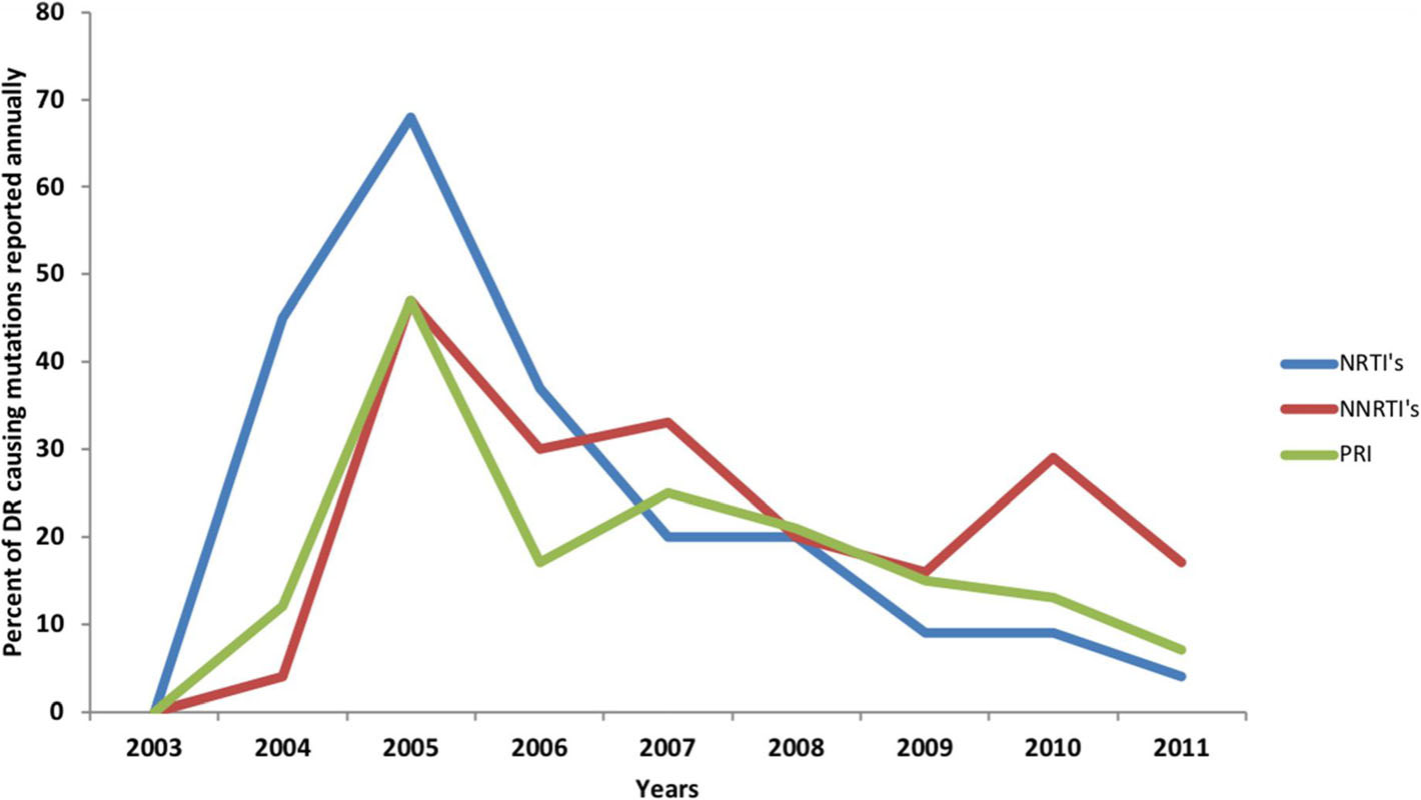
The frequency of reported mutations per group of ART drugs from 2003 to 2016. The number of new mutations reported for every drug in the three major ART groups, NNRTI’s (non-nucleoside reverse transcriptase inhibitors), NRTI’s (Reverse transcriptase inhibitors) and PI’s (Protease inhibitors) was combined and plotted

The highest frequency for drug resistance in our cohort studies was reported to the NRTI’s (68%) followed by (47%) for both NNRTI’s and PRI’s, as seen in Fig. 1.

**Fig. 2:**
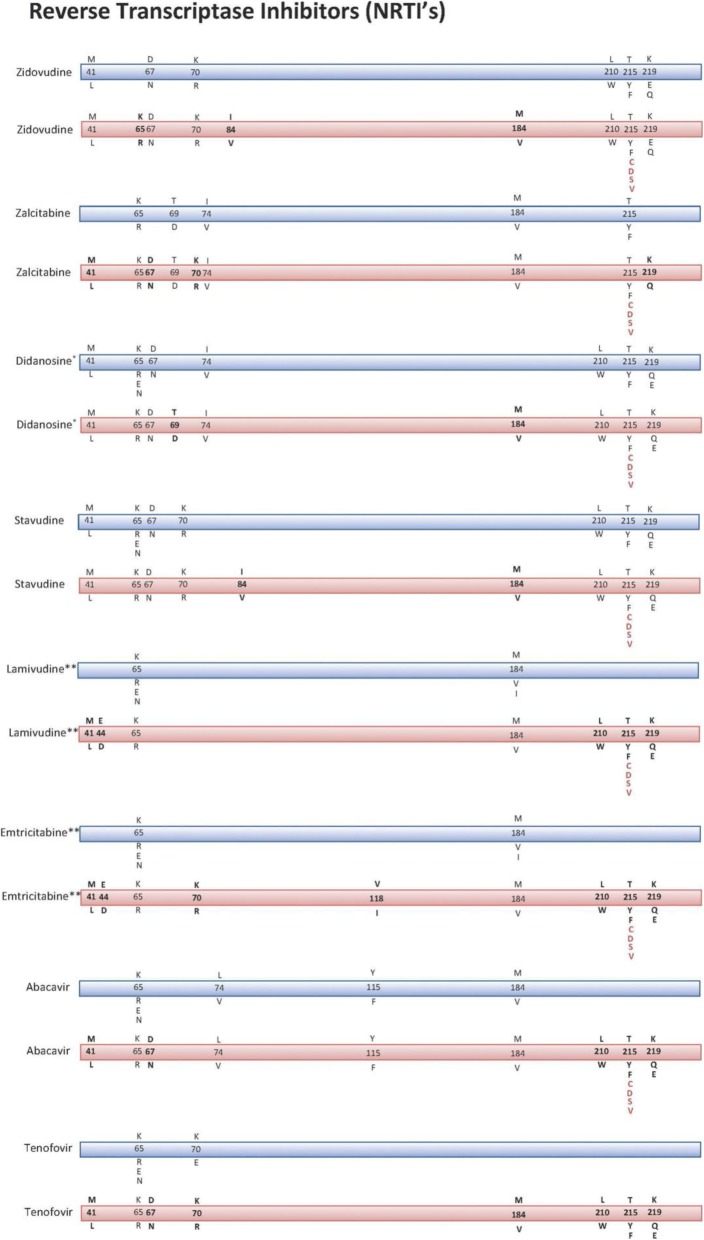
Comparison of the resistance causing mutations reported internationally and at KFSH&RC in the NNRTI’s (non-nucleoside reverse transcriptase inhibitors) group of ART. The Blue ribbon represents the World Reported mutations, while the Pink ribbons are the mutations reported at KFSH& RC reported mutations. Bold letter represents the world Reported mutations but with a different drug at KFSH&RC (different profile of mutations), while the Red represents the Novel mutations which were never previously reported. ^*^M184 and K70R, were reported in both the world report and KFSH&RC, they do not contribute to development of drug resistance. ^**^Reported mutations at KFSH&RC: E44D and V188I are reported in general to have a limited accessory role in increasing resistance

Overall the K103 N was the main and most common mutation detected within the NNRTIs group of drugs. Other Mutations at positions; 98, 100, 101, 103, 106, 108, 138, 179, 181, 188, 190, 225, 227 and 230 have also been detected. Interestingly, mutation I88H, K101Q, E135G, V179E and F227 L are novel mutations reported with our patients’ cohort which were never reported previously. Furthermore, mutations V106A, V108I, Y181C, Y88L, G190A and P225H have been reported in our patients. Figure 2, shows the international reported mutations and their profile in comparison to the resistance causing mutations reported in our cohort of patient sample at KFSH&RC.

**Fig. 3:**
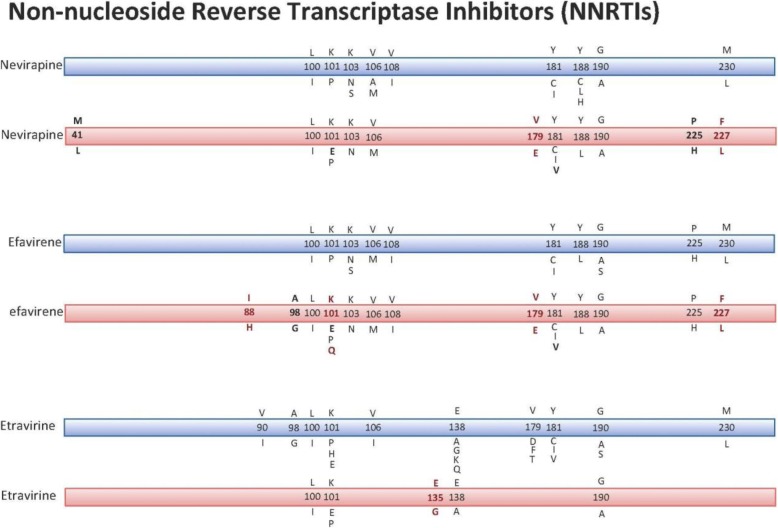
Comparison of the resistance causing mutations reported internationally and at KFSH&RC in the NRTI’s (nucleoside reverse transcriptase inhibitors) group of ART. The Blue ribbon represents the World Reported mutations, while the Pink ribbons are the mutations reported at KFSH& RC reported mutations. Bold letter represents the world Reported mutations but with a different drug at KFSH&RC (different profile of mutations), while the Red represents the Novel mutations which were never previously reported

Resistance to NRTI’s especially AZT was found to be induced by a range of mutations in RT including M41 L, K65R/E/N, D67N, K70R, I74V, I84V, M184 L, L210 W, T215Y/F, and K219E which were all present in our patients. Additionally, a number of resistance causing novel mutations were also observed, including, T215C/D/S/V. Figure 3, demonstrate previously reported mutations in comparison with our reported mutations, which indicates a different profile and reported novel mutations within the NRTI’s.

**Fig. 4:**
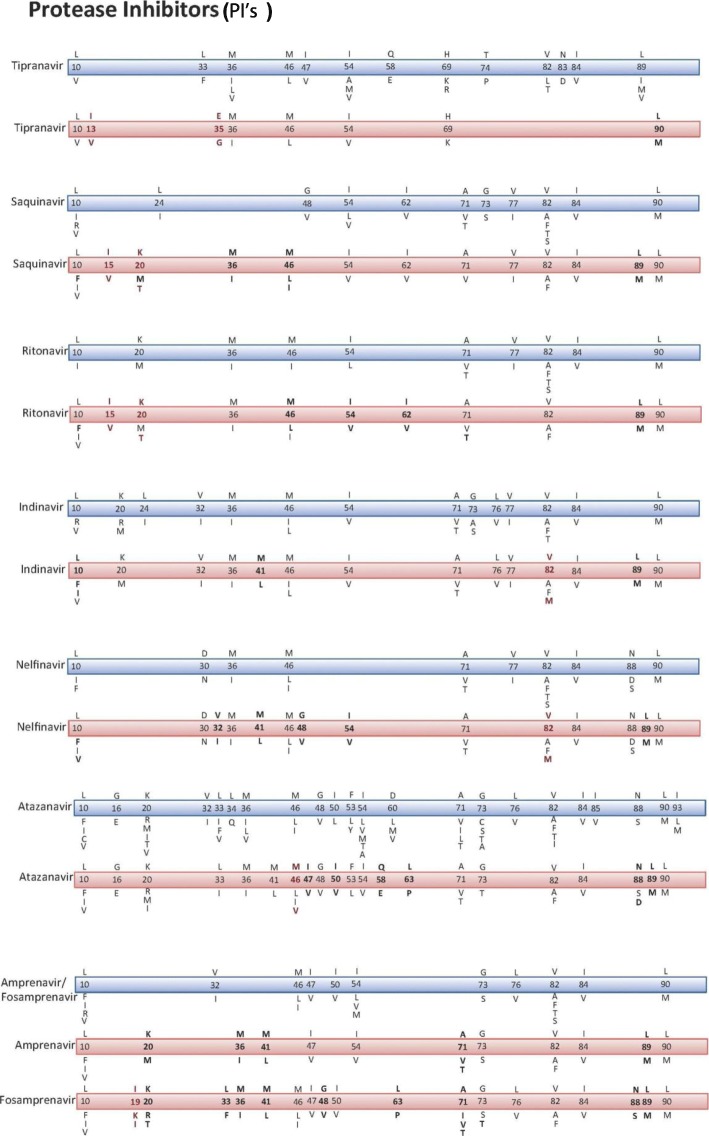
Comparison of the resistance causing mutations reported internationally and at KFSH&RC in the PI’s (Protease inhibitors) group of ART. The Blue ribbon represents the World Reported mutations, while the Pink ribbons are the mutations reported at KFSH& RC reported mutations. Bold letter represents the world Reported mutations but with a different drug at KFSH&RC (different profile of mutations), while the Red represents the Novel mutations which were never previously reported

**Fig. 5:**
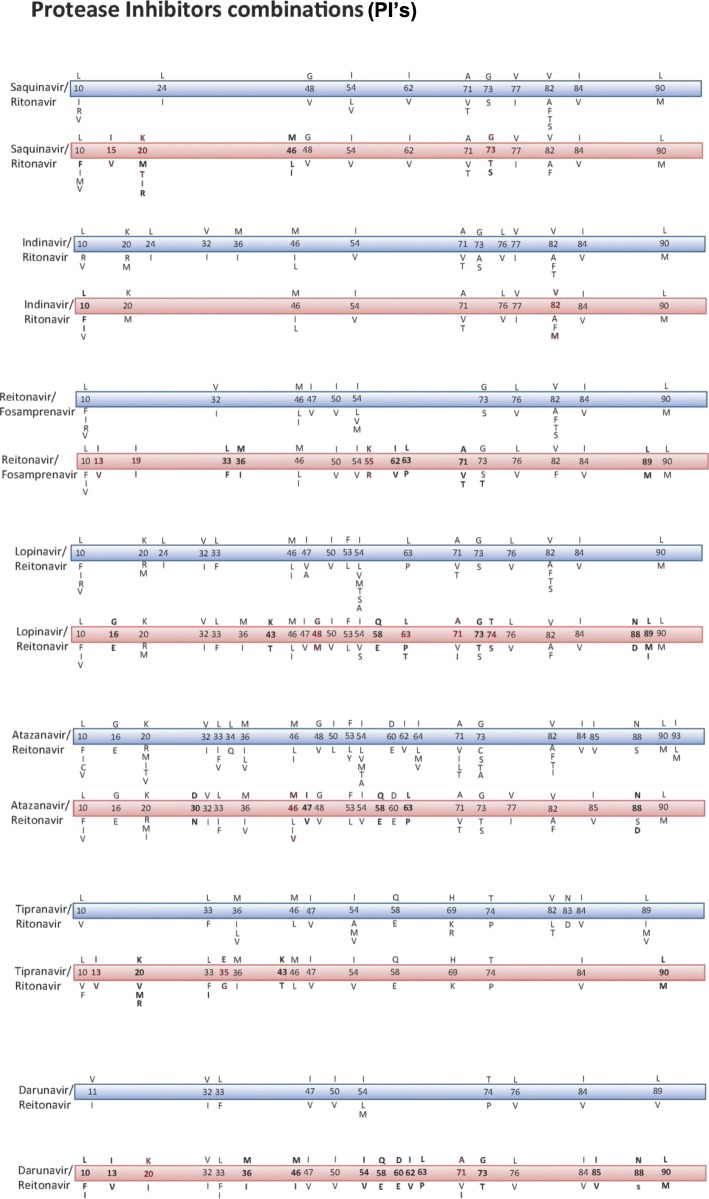
Comparison of the resistance causing mutations reported internationally and at KFSH&RC in the PI’s (Protease inhibitors) combinations. The Blue ribbon represents the World Reported mutations, while the Pink ribbons are the mutations reported at KFSH& RC reported mutations. Bold letter represents the world Reported mutations but with a different drug at KFSH&RC (different profile of mutations), while the Red represents the Novel mutations which were never previously reported

The use of PI’s alone also yielded a number of mutations, similar to what is reported internationally, as seen in Fig. 4. Additionally, when a combination of PI’s are used in therapy protocol, mutations at codon 82, V82A/T/F/S, was reported in our patients receiving treatment with indinavir and ritonavir. Mutation D30N is in our cohort patients receiving a combined therapy of atazanavir and reitonavir. A comparison of the mutations reported in the sample from KFSH&RC and the internationally reported mutations is listed in Fig. 5.

## Discussion

Generally the K103 N was the main mutation detected within the NNRTIs group of drugs. K103 N is the most common resistance mutation that emerges during failure of an NNRTI-containing regimen [10], which is also the case with our patients, where this mutation was detected in 100% of patients treated with either efavirenz or nevirapine. A major limitation for treatment with NNRTIs is the rapid development of drug-resistant mutants. A single mutation may cause a high-level drug resistance to NNRTIs and can confer significant cross-resistance among all NNRTIs [11–13]. Other Mutations at positions; 98, 100, 101, 103, 106, 108, 138, 179, 181, 188, 190, 225, 227 and 230 have also been detected, most of these mutations have been shown to confer resistance in NNRTIs [14–16]. Interestingly, mutation I88H, K101Q, E135G, V179E and F227 L are novel mutations which were never reported previously. These mutations confers high-level cross-resistance to all currently available NNRTIs [8, 17, 18]. The mutations V106A, V108I, Y181C, Y88L, G190A and P225H have been reported in our patients. There is evidence that the following mutations V106A, Y181C and G190A have been observed in early virological failure.

Resistance to NRTI’s, specially AZT was found to be induced by a range of mutations in RT including M41 L, K65R/E/N, D67N, K70R, I74V, I84V, M184 L, L210 W, T215Y/F, and K219E as previously reported in previous studies and annual reports [13, 19]. Furthermore, a number of resistance causing novel mutations were also reported, they include, T215C/D/S/V. Although the mutations detected are similar to what was earlier reported [13], some of them are associated with different drugs from what was earlier reported, leading to presenting a different profile of mutations. This difference includes mutation L210 W, which was reported with didanosine and stavudine only, in our analysis the same mutation is also seen with lamivudine, emtricitabine, abacavir and tenogovir. Mutations T215Y/F and K219 also are present in association with lamivudine, emtricitabine, abacavir and tenofovir, which were not reported previously. Additionally, a number of mutations that were reported internationally but are of minimal or no role in the development of resistance were also seen in our sample, including mutation M184 V and K70R. Furthermore, mutations E44D and V188I were also present in our sample and reported internationally, these mutations in general have a limited accessory role in increasing resistance in general [8, 13].

Mutations in the protease gene which is mediated by amino acid substitutions results in reducing the binding of the inhibitor to the protease leading to drug resistance to PI’s [20–22]. The most common mutated codons; that are related to PI’s drug resistance are: L10I, K20 T, M36I, M41 L, M46I, I54V, I62V, A71V, and L90 M. Minor mutations were found in the protease gene, including L10F, L10 V, I15V, K20 M, D30N, I47V, I62V, V77I, V82A, V82F, I84V, L63P, H69K, A71T, G73SL76V, V82I, L89 M and I93L. Additionally, a number of novel mutations were detected in our patients, which was not reported previously such as I13V, I15V, E35G, M46 V, and V82 M. The L90 M codon was found to be associated with resistance to all seven PIs in cohort studies. In addition the L90 M has been associated with resistance in vitro therefore conferring broad cross-class resistance [23–25].

Additionally, when combinations of PI’s are used in therapy protocol, another set of mutations appear, both novel and reported. For example, mutation at codon 82, V82A/T/F/S, is reported in patients receiving treatment with indinavir and ritonavir [21] and also in our patients. Mutation D30N is observed in patients receiving nelfinaviras was reported [26], additionally the same mutation was observed in our cohort patients receiving a combined therapy of atazanavir and reitonavir. Polymorphisms at codons 10, 20, 36, 63, 71, 77, and 93 have been described as lower rate frequency mutations. However, these mutations do not cause drug resistance in isolation but when present with other primary PI mutations [13, 27].

It has been shown that mutations at codon 10, 63, or 71 compensated for a reduced viability of HIV-1 variants with a primary resistance mutation at codon 82 [22]. High-level resistance caused by the accumulation of primary and secondary mutations can lead to clinically significant reductions in drug susceptibility [28]. A mutation at codon 63 has been shown to be the most polymorphic mutation in the protease gene [29]. L63P is found about 45% in untreated individuals and approximately 10% carried other polymorphisms at this codon including L63S/T/S/Q/N [30].

## Conclusion

With the expansion and the availability of different classes of ARV, virological suppression has been mostly achievable. Yet, with the continuous administration of HAART therapy, the development of ARV resistance is the major complication and challenge, in addition to other challenges such as patient compliance and development of ARV therapy toxicity [31–33]. Currently the WHO publishes reports which addresses and lists all the HIV resistance causing mutations reported at a global level [34], furthermore, the Office of AIDS Research Advisory Council (OARAC) publishes periodical guidelines for HIV therapy in all groups and populations based on all the reported and published cases [6]. Additionally, some countries develop their own therapy guidelines also based on a number of factors such as the population, the reports and the availability of the drugs [35–38], in addition to KFSH&RC. These guidelines are updated annually, but in the end, it’s not set rules, but rather a set of recommendations to be followed if applicable only and based on the individual patient profile and situation, in addition to the availability of the ARV on the time of diagnosis and initiation of therapy. The aim of these guidelines is to provide the maximum virological suppression with minimum toxicity and drug resistance development. Effort has been expended to use different drug regimens in order to reduce the development of resistance.

Despite these efforts, drug-resistant viruses are increasing in the recent years in the HIV-infected population and this is caused by the rapid replication rate of the virus and its characteristic genetic adaptation capabilities [39–45]. Based on the genotypic polymorphisms studies these emerging resistances have been labelled as either primary or secondary resistance mutations. Infection of individuals with drug-resistant alternates of the virus has therapeutic failure consequences for the individual, and also has a domino effect on any future therapy of this epidemic on population basis [2, 45].

Antiretroviral therapy failure is well documented and is characterized by the increase in viral replication and load. There are various causes leading to this failure, which is mainly caused by sub-therapeutic drug levels due to drug interactions, poor compliance, or poor absorption, or not following the proper protocol [4, 33]. Thus its concluded that the development of resistance to antiretroviral therapy is a major contributor to drug failure [7, 9].

**Fig. 6:**
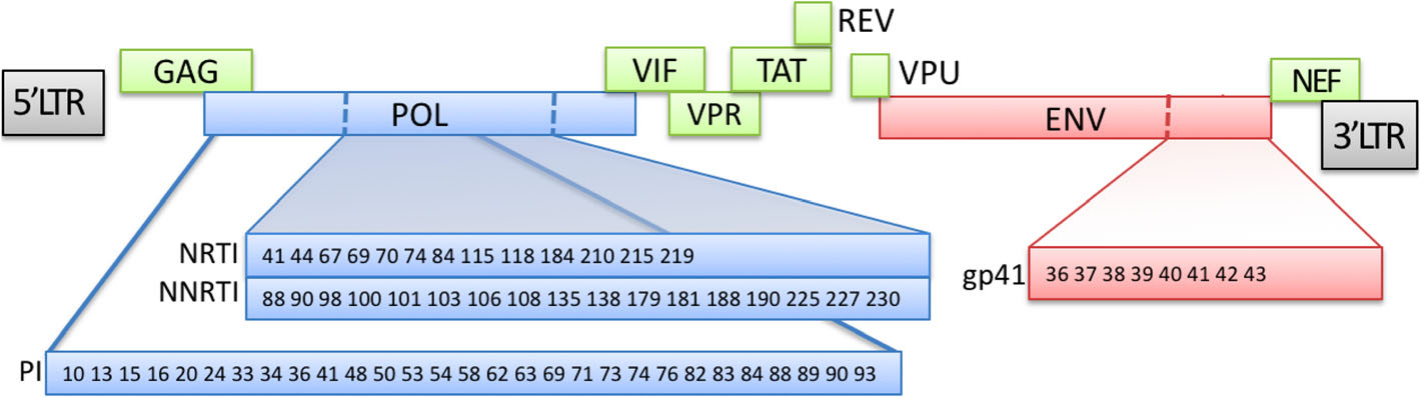
List of all mutations reported internationally as well as the ones reported in KFSH&RC on the HIV-1 open reading frame. Shows the location of the mutations on the POL gene of HIV-1. Additionally, it shows the drug targets on the same gene and the resistance causing mutations for both internationally reported (black) and novel mutations (red)

Additionally, different backgrounds of the patients and their location all contribute to the developing of resistance causing mutations [32]. Periodical reporting of these mutations in the Kingdom of Saudi Arabia (KSA), for example, will contribute to better therapy and outcome for the patient. The presence of novel mutations, which were not reported previously in the international annual report [13, 34], highlights the importance of these periodic reports from this area of the globe, since it is already done in a number of other areas such as the sub-Saharan African area [38], and India [37], in addition to specific WHO associated reports such as the WHO / Bhutan 2008 report (WHO, 2008). Figure 6 summarizes these internationally reported mutations in addition to the novel ones reported from our sample at KFSH&RC.

The prevalence of primary or transmitted HIV drug resistance to all of the drugs and drug classes that were evaluated in this study was 66% and 34% respectively. These findings provide a useful background for antiretroviral therapy in KSA and contribute reference data for the surveillance of HIV drug resistance around the world. Additionally, the frequency of the reported mutations and the novel ones, as well as the outcome will be an interesting future study, additionally, reporting the patient behavior towards therapy and their compliance and its correlation with the frequency of development of both types of mutations would also be interesting. There are limited HIV drug resistance surveillance reports done, especially in KSA, this paper is a preliminary retrospective report and analysis of drug resistance mutations among our HIV patients. Identifying these resistance causing mutations will guide us to establish an in house diagnostic assay for screening of these possible DRM as a diagnostic tool for the HIV clinic and therefore to carefully monitor these resistant strains.

## Abbreviations

ART: Anti-retroviral therapy
ARV: Anti-retroviral drugs
DRM: Drug resistant mutations
FI: Fusion inhibitors
HAART: Highly active antiretroviral therapy
II: Integrase inhibitors
KFSH&RC: King Faisal Specialist Hospital and Research Center
KSA: Kingdom of Saudi Arabia
NNRTIs: Non-nucleoside reverse transcriptase inhibitors
NRTIs: Nucleoside reverse transcriptase inhibitors
PIs: Protease inhibitors

## References

[1] Palella FJ, Armon C, Buchacz K, Cole SR, Chmiel JS, Novak RM, Wood K, Moorman AC, Brooks JT (2009). The association of HIV susceptibility testing with survival among HIV-infected patients receiving antiretroviral therapy: a cohort study. Ann Intern Med.

[2] Ammaranond P, Sanguansittianan S (2012). Mechanism of HIV antiretroviral drugs progress toward drug resistance. Fundam Clin Pharmacol.

[3] Tang MW, Shafer RW (2012). HIV-1 antiretroviral resistance: scientific principles and clinical applications. Drugs.

[4] Zhang XQ (2015). The newest developments of the study on anti-HIV drugs. Yao xue xue bao = Acta pharmaceutica Sinica.

[5] WHO. Consolidated strategic information guidelines for HIV in the health sector: WHO; 2015. Print 26110192

[6] DHHS DoHaHS: Panel on Antiretroviral Guidelines for Adults and Adolescents. Guidelines for the use of antiretroviral agents in HIV-1-infected adults and adolescents. . In*.*; 2015.

[7] Nosik MN (2014). A problem of the HIV drug resistance. Vopr Virusol.

[8] Sluis-Cremer N, Wainberg MA, Schinazi RF. Resistance to reverse transcriptase inhibitors used in the treatment and prevention of HIV-1 infection. Future Microbiol. 2015;10(11):1773–82. 10.2217/fmb.15.106 PMC4813512 26517190

[9] Roche M, Borm K, Flynn JK, Lewin SR, Churchill MJ, Gorry PR. Molecular gymnastics: mechanisms of HIV-1 resistance to CCR5 antagonists and impact on virus phenotypes. Curr Top Med Chem. 2015; 10.2174/1568026615666150901114724 26324043

[10] Hanna GJ, Johnson VA, Kuritzkes DR, Richman DD, Brown AJ, Savara AV, Hazelwood JD, D'Aquila RT (2000). Patterns of resistance mutations selected by treatment of human immunodeficiency virus type 1 infection with zidovudine, didanosine, and nevirapine. J Infect Dis.

[11] Deeks SG (2001). International perspectives on antiretroviral resistance. Nonnucleoside reverse transcriptase inhibitor resistance. J Acquir Immune Defic Syndr.

[12] Johnson VA, Brun-Vezinet F, Clotet B, Gunthard HF, Kuritzkes DR, Pillay D, Schapiro JM, Richman DD (2007). Update of the drug resistance mutations in HIV-1: 2007. Top HIV Med.

[13] Wensing AM, Calvez V, Gunthard HF, Johnson VA, Paredes R, Pillay D, Shafer RW, Richman DD (2014). 2014 update of the drug resistance mutations in HIV-1. Top Antivir Med.

[14] Ren J, Nichols CE, Stamp A, Chamberlain PP, Ferris R, Weaver KL, Short SA, Stammers DK (2006). Structural insights into mechanisms of non-nucleoside drug resistance for HIV-1 reverse transcriptases mutated at codons 101 or 138. FEBS J.

[15] Ren J, Nichols CE, Chamberlain PP, Weaver KL, Short SA, Chan JH, Kleim JP, Stammers DK (2007). Relationship of potency and resilience to drug resistant mutations for GW420867X revealed by crystal structures of inhibitor complexes for wild-type, Leu100Ile, Lys101Glu, and Tyr188Cys mutant HIV-1 reverse transcriptases. J Med Chem.

[16] Das K, Sarafianos SG, Clark AD, Boyer PL, Hughes SH, Arnold E (2007). Crystal structures of clinically relevant Lys103Asn/Tyr181Cys double mutant HIV-1 reverse transcriptase in complexes with ATP and non-nucleoside inhibitor HBY 097. J Mol Biol.

[17] Hsiou Y, Ding J, Das K, Clark AD, Boyer PL, Lewi P, Janssen PA, Kleim JP, Rosner M, Hughes SH (2001). The Lys103Asn mutation of HIV-1 RT: a novel mechanism of drug resistance. J Mol Biol.

[18] D'Aquila RT, Schapiro JM, Brun-Vezinet F, Clotet B, Conway B, Demeter LM, Grant RM, Johnson VA, Kuritzkes DR, Loveday C (2002). Drug resistance mutations in HIV-1. Top HIV Med.

[19] Shafer RW (2002). Genotypic testing for human immunodeficiency virus type 1 drug resistance. Clin Microbiol Rev.

[20] Erickson JW, Gulnik SV, Markowitz M (1999). Protease inhibitors: resistance, cross-resistance, fitness and the choice of initial and salvage therapies. AIDS (London, England).

[21] Martinez-Cajas JL, Wainberg MA (2007). Protease inhibitor resistance in HIV-infected patients: molecular and clinical perspectives. Antivir Res.

[22] Sune C, Brennan L, Stover DR, Klimkait T (2004). Effect of polymorphisms on the replicative capacity of protease inhibitor-resistant HIV-1 variants under drug pressure. Clin Microbiol Infect.

[23] Falloon J, Piscitelli S, Vogel S, Sadler B, Mitsuya H, Kavlick MF, Yoshimura K, Rogers M, LaFon S, Manion DJ (2000). Combination therapy with amprenavir, abacavir, and efavirenz in human immunodeficiency virus (HIV)-infected patients failing a protease-inhibitor regimen: pharmacokinetic drug interactions and antiviral activity. Clin Infect Dis.

[24] Hertogs K, Bloor S, Kemp SD, Van den Eynde C, Alcorn TM, Pauwels R, Van Houtte M, Staszewski S, Miller V, Larder BA (2000). Phenotypic and genotypic analysis of clinical HIV-1 isolates reveals extensive protease inhibitor cross-resistance: a survey of over 6000 samples. AIDS (London, England).

[25] Para MF, Glidden DV, Coombs RW, Collier AC, Condra JH, Craig C, Bassett R, Leavitt R, Snyder S, McAuliffe V (2000). Baseline human immunodeficiency virus type 1 phenotype, genotype, and RNA response after switching from long-term hard-capsule saquinavir to indinavir or soft-gel-capsule saquinavir in AIDS clinical trials group protocol 333. J Infect Dis.

[26] Tupinambas U, Aleixo A, Greco D (2005). HIV-1 genotypes related to failure of nelfinavir as the first protease inhibitor treatment. Braz J Infect Dis.

[27] Mammano F, Trouplin V, Zennou V, Clavel F (2000). Retracing the evolutionary pathways of human immunodeficiency virus type 1 resistance to protease inhibitors: virus fitness in the absence and in the presence of drug. J Virol.

[28] Kempf DJ, Isaacson JD, King MS, Brun SC, Xu Y, Real K, Bernstein BM, Japour AJ, Sun E, Rode RA (2001). Identification of genotypic changes in human immunodeficiency virus protease that correlate with reduced susceptibility to the protease inhibitor lopinavir among viral isolates from protease inhibitor-experienced patients. J Virol.

[29] Pieniazek D, Rayfield M, Hu DJ, Nkengasong J, Wiktor SZ, Downing R, Biryahwaho B, Mastro T, Tanuri A, Soriano V (2000). Protease sequences from HIV-1 group M subtypes A-H reveal distinct amino acid mutation patterns associated with protease resistance in protease inhibitor-naive individuals worldwide. HIV Variant Working Group. AIDS (London, England).

[30] Kantor R, Machekano R, Gonzales MJ, Dupnik K, Schapiro JM, Shafer RW (2001). Human immunodeficiency virus reverse transcriptase and protease sequence database: an expanded data model integrating natural language text and sequence analysis programs. Nucleic Acids Res.

[31] Nelson AG, Zhang X, Ganapathi U, Szekely Z, Flexner CW, Owen A, Sinko PJ. Drug delivery strategies and systems for HIV/AIDS pre-exposure prophylaxis and treatment. J Control Release. 2015; 10.1016/j.jconrel.2015.08.042 PMC4879940 26315816

[32] Uliukin IM, Bolekhan VN, Iusupov VV, Bulan'kov Iu I, Orlova ES (2015). Problems of early detection of HIV infection, medical and psychological support of HIV-infected soldiers. Voenno-meditsinskii zhurnal.

[33] de Martino M, Galli L, Chiappini E: Perinatal human immunodeficiency virus type-1 in the 21st century: new challenges in treatment and health care organization. Pediatr Infect Dis J 2015, 34(5 Suppl 1):S1–S2. 10.1097/INF.0000000000000658 25894972

[34] WHO (2012). WHO HIV Resistance report 2012.

[35] Antinori A, Di Biagio A, Marcotullio S, Andreoni M, Chirianni A, d'Arminio Monforte A, Galli M, Mazzotta F, Mussini C, Puoti M (2017). Italian guidelines for the use of antiretroviral agents and the diagnostic-clinical management of HIV-1 infected persons. Update 2016. New Microbiol.

[36] Kouakou-Siransy G, Horo K, Effo E, Kamenan A, N'Guessan-Irie G, Boko-Kouassi A, Aka-Anguy E, Kakou HD (2015). Pharmacotherapeutic aspect of antibiotic therapy during acute community-acquired pneumonia in adults at the University Hospital of Cocody (Abidjan). Int J Clin Pharmacol Ther.

[37] Seenivasan S, Vaitheeswaran N, Seetha V, Anbalagan S, Karunaianantham R, Swaminathan S (2015). Outcome of prevention of parent-to-child transmission of HIV in an urban population in southern India. Indian Pediatr.

[38] Ssemwanga D, Lihana RW, Ugoji C, Abimiku A, Nkengasong J, Dakum P, Ndembi N (2015). Update on HIV-1 acquired and transmitted drug resistance in Africa. AIDS Rev.

[39] Weinstock H, Respess R, Heneine W, Petropoulos CJ, Hellmann NS, Luo CC, Pau CP, Woods T, Gwinn M, Kaplan J (2000). Prevalence of mutations associated with reduced antiretroviral drug susceptibility among human immunodeficiency virus type 1 seroconverters in the United States, 1993-1998. J Infect Dis.

[40] Havlir DV, Hellmann NS, Petropoulos CJ, Whitcomb JM, Collier AC, Hirsch MS, Tebas P, Sommadossi JP, Richman DD (2000). Drug susceptibility in HIV infection after viral rebound in patients receiving indinavir-containing regimens. Jama.

[41] Harrigan PR, Hogg RS, Dong WW, Yip B, Wynhoven B, Woodward J, Brumme CJ, Brumme ZL, Mo T, Alexander CS (2005). Predictors of HIV drug-resistance mutations in a large antiretroviral-naive cohort initiating triple antiretroviral therapy. J Infect Dis.

[42] Aleman S, Soderbarg K, Visco-Comandini U, Sitbon G, Sonnerborg A (2002). Drug resistance at low viraemia in HIV-1-infected patients with antiretroviral combination therapy. AIDS (London, England).

[43] Idemyor V (2002). Human immunodeficiency viruses and drug therapy: resistance and implications for antiretroviral therapy. Pharmacotherapy.

[44] Tang MW, Shafer RW (2012). HIV-1 antiretroviral resistance: scientific principles and clinical applications. Drugs.

[45] Santoro MM, Perno CF. HIV-1 genetic variability and clinical implications. ISRN Microbiology. 2013;2013 10.1155/2013/481314 PMC3703378 23844315

